# Characterization of avian influenza virus attachment patterns to human and pig tissues

**DOI:** 10.1038/s41598-018-29578-1

**Published:** 2018-08-15

**Authors:** Per Eriksson, Cecilia Lindskog, Ebbe Engholm, Ola Blixt, Jonas Waldenström, Vincent Munster, Åke Lundkvist, Björn Olsen, Elsa Jourdain, Patrik Ellström

**Affiliations:** 1 Zoonosis Science Centre, Department of Medical Biochemistry and Microbiology (IMBIM), Uppsala University, Uppsala, SE 751 23 Sweden; 2 Department of Immunology, Genetics and Pathology, Science for Life Laboratory, Uppsala University, Uppsala, SE 751 85 Sweden; 3 Department of Chemistry, Chemical Biology, University of Copenhagen, Frederiksberg C, DK 1871 Denmark; 4 Centre for Ecology and Evolution in Microbial Model Systems, Linnaeus University, Kalmar, SE 392 31 Sweden; 5 Laboratory of Virology, Rocky Mountain Laboratories, National Institute of Allergy and Infectious Diseases, National Institutes of Health, Hamilton, Montana, 59840 USA; 6 Zoonosis Science Centre, Department of Medical Sciences, Uppsala University, Uppsala, SE 751 23 Sweden; 7 UMR0346 - EPIA, INRA, VetAgro Sup, Saint Genès Champanelle, FR 63122 France

## Abstract

Wild birds of Anseriformes and Charadriiformes are natural reservoirs of influenza A viruses (IAVs). Occasionally, IAVs transmit and adapt to mammalian hosts, and are maintained as epidemic strains in their new hosts. Viral adaptions to mammalian hosts include altered receptor preference of host epithelial sialylated oligosaccharides from terminal α2,3-linked sialic acid (SA) towards α2,6-linked SA. However, α2,3-linked SA has been found in human respiratory tract epithelium, and human infections by avian IAVs (AIVs) have been reported. To further explore the attachment properties of AIVs, four AIVs of different subtypes were investigated on human and pig tissues using virus histochemistry. Additionally, glycan array analysis was performed for further characterization of IAVs’ receptor structure tropism. Generally, AIV attachment was more abundant to human tissues than to pig tissues. The attachment pattern was very strong to human conjunctiva and upper respiratory tract, but variable to the lower respiratory tract. AIVs mainly attached to α2,3-linked SA, but also to combinations of α2,3- and α2,6-linked SA. The low attachment of these AIV isolates to pig tissues, but high attachment to human tissues, addresses the question whether AIVs in general require passage through pigs to obtain adaptions towards mammalian receptor structures.

## Introduction

Global circulation of influenza A viruses (IAVs) in animals, especially avian IAVs (AIVs) in birds, poses a risk for development of IAVs pathogenic to humans^1^. Wild birds of the Anseriformes and Charadriiformes orders (e.g. waterfowl and shorebirds) are the main natural reservoir of AIVs^1,2^, potentially along with other avian species that have been less frequently investigated. However, AIVs do not commonly transmit directly to humans and are thought to require sequential adaption to be efficiently transmitted among humans^3^. The described human IAV pandemics are believed to originate from low pathogenic AIVs (LPAIVs) from wild birds, which have been transmitted from wild bird hosts to domestic fowl and further to pigs, or directly from wild birds to pigs^4^. In coinfected pigs, IAV of avian and mammalian type can reassort and produce novel IAVs adapted for respiratory tract infection in mammals, including humans^5^. Pigs are regarded as a “mixing vessel” for IAV, due to their dual susceptibility to avian and human IAVs^6,7^. Hallmarks of avian vs. human IAVs include AIVs binding to α2,3-linked *N*-acetylneuraminic acid (SA) and human IAVs binding to α2,6-linked SA for host cell attachment^8,9^. Moreover, AIVs predominantly target gastrointestinal tissues in their natural hosts, whereas human-adapted IAVs are respiratory pathogens in humans^1,10^. Further studies have refined the knowledge on receptor tropism of IAVs^11^. For instance, Neu5Acα2–6Galβ1-4GlcNAc (6′SLN) was reported as the essential receptor structure for human H3N2 IAV binding^12^. Additionally, acetylation of Gal was tolerated, as well as sulfation of the GlcNAc residue. In contrast, it was reported that AIVs isolated from Anseriformes hosts prefer Neu5Acα2-3Galβ1-4GlcNAc (3′SLN), whereas highly pathogenic poultry H5N1 viruses had an increased affinity for Su-3′SLN^13^. AIV receptor tropism has been reported to follow host phylogeny of ducks, gulls and chickens^14^. Duck viruses (H1, H2, H3, H4, H5, H9, H11, H12 and H14) had the highest affinity for Neu5Acα2-3Galβ1-3GalNAc (3′STF). Gull viruses (H4, H5, H6, H13 and H14) preferred Neu5Acα2-3Galβ1-4(Fucα1-3)GlcNAc (SLe^x^). Whereas, chicken viruses (H5 and H7) preferred Neu5Acα2-3Galβ1-4(Fucα1-3)(6-O-HSO_3_)GlcNAc (Su-SLe^x^). In another study of mallard IAVs of various subtypes (H1, H2, H4 and H10), the highest viral affinity was observed for Neu5Acα2-3Galβ1-3GlcNAc (SLe^c^)^13^. In summary, these studies suggested that human IAVs have the highest observed affinity for 6′SLN, whereas AIVs have the highest affinity for 3′SLN or 3′STF with slight modifications depending on the avian host species^13–15^. The sialylated glycan structures utilized by IAVs for host cell attachment are displayed at the cell surface of the host cell. Thus, the susceptibility for IAV infection is dependent on the host cell surface receptor expression^16^. Commonly, lectins from *Maackia amurensis* (MAA) and *Sambucus nigra* (SNA) have been used to visualize Neu5Acα2-3Gal and Neu5Acα2-6Gal(NAc), respectively. For IAVs, α2-6-linked SA is commonly referred to as the “human receptor” and is abundantly found in the human respiratory tract^3^, while α2-3-linked SA is found in the intestinal tract of birds and termed the “avian receptor”. It was originally believed that humans only express SA of the “human receptor” type and birds exclusively express SA of the “avian receptor” type. In pigs, SA of both avian and human receptor types is displayed, rendering this species susceptible also for avian strains^7^. However, it is now known that humans display high numbers of “avian receptor” type SA in the respiratory tract^17^. Additionally, there is evidence for presence of the “human receptor” in tracheal epithelia of several bird species^18,19^.

Attachment to host epithelial cells initiates IAV infection^20^. Therefore, studying the pattern of virus attachment (PVA) to host tissues is an important tool to predict IAVs’ host, tissue and cell tropisms^10^. Increased numbers of reports on human infection by AIVs have provided proof that AIVs can infect humans^21,22^. Several recent studies have shown that LPAIVs are capable to attach to human tissues, including human seasonal H3N2 and pandemic H1N1 viruses, avian H7N9 virus, as well as highly pathogenic AIVs (HPAIV) H5N1 and H7N7 using virus histochemistry^22^. Seasonal and pandemic human-adapted viruses mainly attached to the upper respiratory tract (URT), while the avian H7 viruses attached strongly to all studied tissues, except trachea. The HPAIV H7N7 and H5N1 viruses mainly attached to the lower respiratory tract (LRT). In an earlier study of a mallard (*Anas platyrhynchos*) origin LPAIV H6N1 virus, the virus had weak attachment to human trachea and bronchus and intermediate attachment to bronchioles and alveoli, and less attachment to the pig tissues^10^. Black-headed gull (*Larus ridibundus*) H16N3 virus was shown to attach strongly to tissues of human respiratory tract and eye^23^. Gulls belong to Charadriiformes and have in recent years been identified as an important IAV reservoir in addition to anseriform birds, but largely carry host restricted hemagglutinin subtypes H13 and H16^24,25^. In summary, the existing reports of virus attachment mainly focus on the HA subtypes H1, H3, H5, H6 and H7 and attachment to human or avian tissues, whereas other subtypes (e.g. H12 and H16) are much less explored. The present study aims at providing a comprehensive analysis of human and pig tissues and glycan receptor tropisms of a panel of AIVs of various subtypes of Anseriformes and Charadriiformes origin, together with visualization of the SA receptor distribution in the investigated tissues. The viral panel was assembled to include IAV of various subtypes and host origins to cover different functional and evolutionary differences in the IAV pool, and was analysed on human and pig respiratory tissues due to the nature of IAVs causing respiratory infections in these animals and reports of the pig respiratory tract being susceptible to both avian and human IAVs^10^. Additionally, human eye tissue was included, due to earlier reports of AIVs causing conjunctivitis in humans^21,26^. Finally, human colon was included for comparison, since AIVs usually infect the gastrointestinal tract in most birds^1^.

## Results

### Glycan array

**Figure 1:**
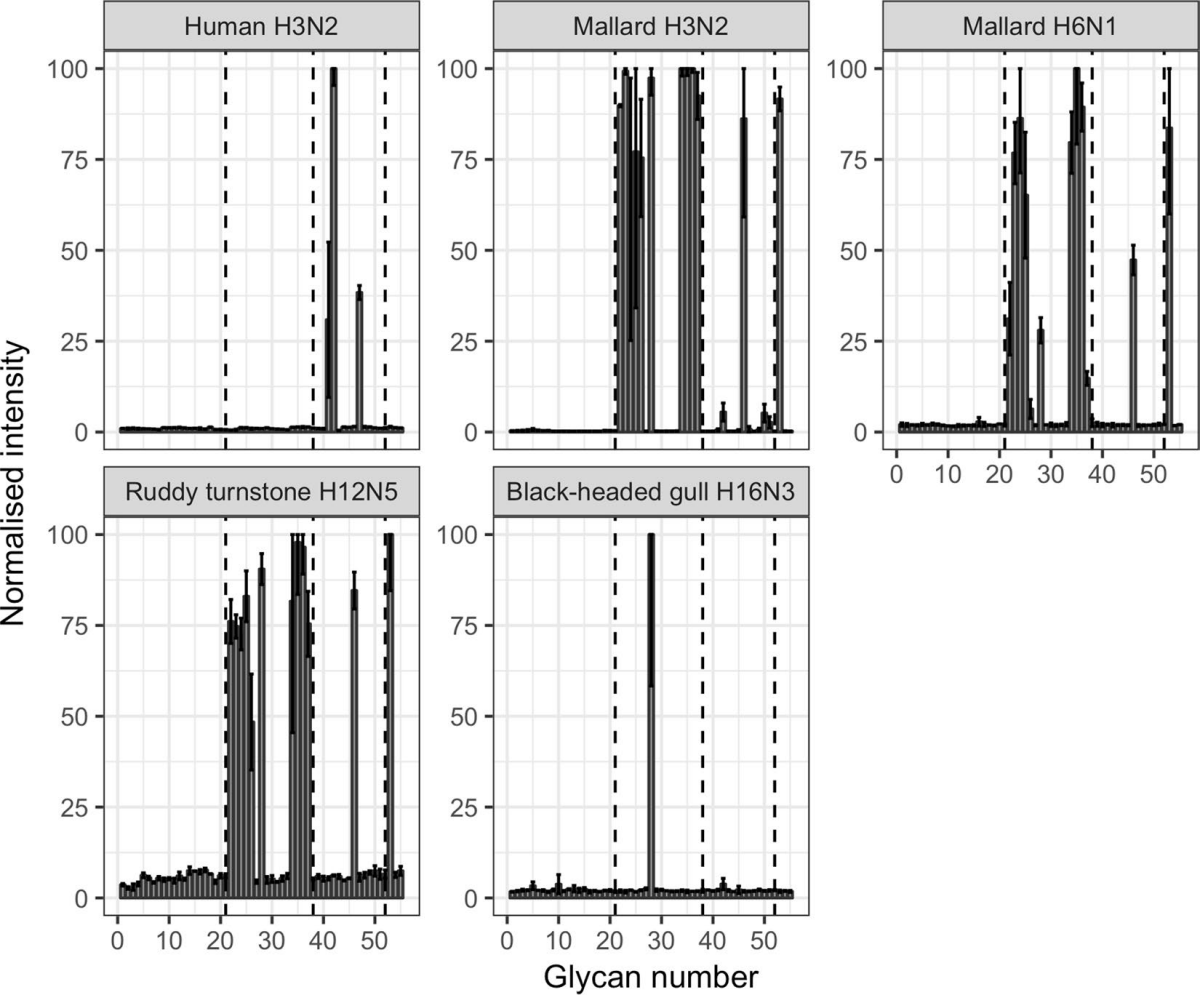
Mean values of triplicate glycan PVA measurements. Error bars show plus/minus one standard deviation. The attachment signals were normalized towards the glycan with the highest virus attachment for each tested virus. Glycans 1–21 do not have any SA, glycans 22–37 all terminate with α2,3-linked SA, glycans 38–52 all terminate with α2,6-linked SA, and glycans 53–55 all carry multiple SAs.

Distinct glycan preferences were observed between the human seasonal IAV and the AIVs. The human seasonal IAV had a well-defined preference for α2,6-linked SAs, whereas all the AIVs attached the most to α2,3-linked SAs (Fig. 1). The structures and abbreviations used for the glycans included in the glycan array, as well as the obtained relative scores are displayed in the supplementary material (Table S1). The human seasonal H3N2 virus had intense attachment to only three different glycans all terminating with α2,6-linked SA: Neu5Acα2-6Galβ1-4GlcNAcβ1-3Galβ1-4GlcNAc (6′SdiLN), Neu5Acα2-6Galβ1-4[6Su]GlcNAc (6Su-6′SLN), and Neu5Acα2-6Galβ1-4GlcNAc (6′SLN).

The mallard H3N2 virus had a broad attachment pattern, but still mainly attached to different α2,3-linked SAs. The most intense attachment was to 3′SLN tri(I)-antennary N-glycan, 3′SLN bi-antennary N-glycan, 3′SLN tri(II)-antennary N-glycan, and single 3′SL to which the mallard H3N2 virus attached equally well. Intense signalling was also observed to 6Su-3′SLN, “chimeric” 3′S[Neu5Acα2-6]TF, branched [6Su]Galβ1-3[Neu5Acα2-6]GalNAc, and 3′STF. Weak attachment was observed to 6′SdiLN and 6′SLN tri-antennary N-glycan. The mallard H6N1 virus preferred α2,3-linked SAs, with the highest attachment to 3′SLN tri-antennary N-glycan, 3′SLN, 3′S[Neu5Acα2-6]TF, 3′SLN bi-antennary N-glycan, 3′SL, 3′STF, [6Su]Galβ1-3[Neu5Acα2-6]GalNAc, “avian receptor”, 6Su-3′SLN, and 3′SLN tetra-antennary N-glycan. The ruddy turnstone H12N5 had the strongest attachment to “chimeric” 3′S[Neu5Acα2-6]TF, 3′SLN tri-antennary N-glycan, 6Su-3′SLN, [6Su]Galβ1-3[Neu5Acα2-6]GalNAc, 3′STF, 3′SLN bi-antennary N-glycan, “avian receptor”, 3′SLN tetra-antennary N-glycan, 3′SL, and 3′SLN. The black-headed gull H16N3 virus only attached to 6Su-3′SLN.

### Virus and lectin histochemistry

**Figure 2:**
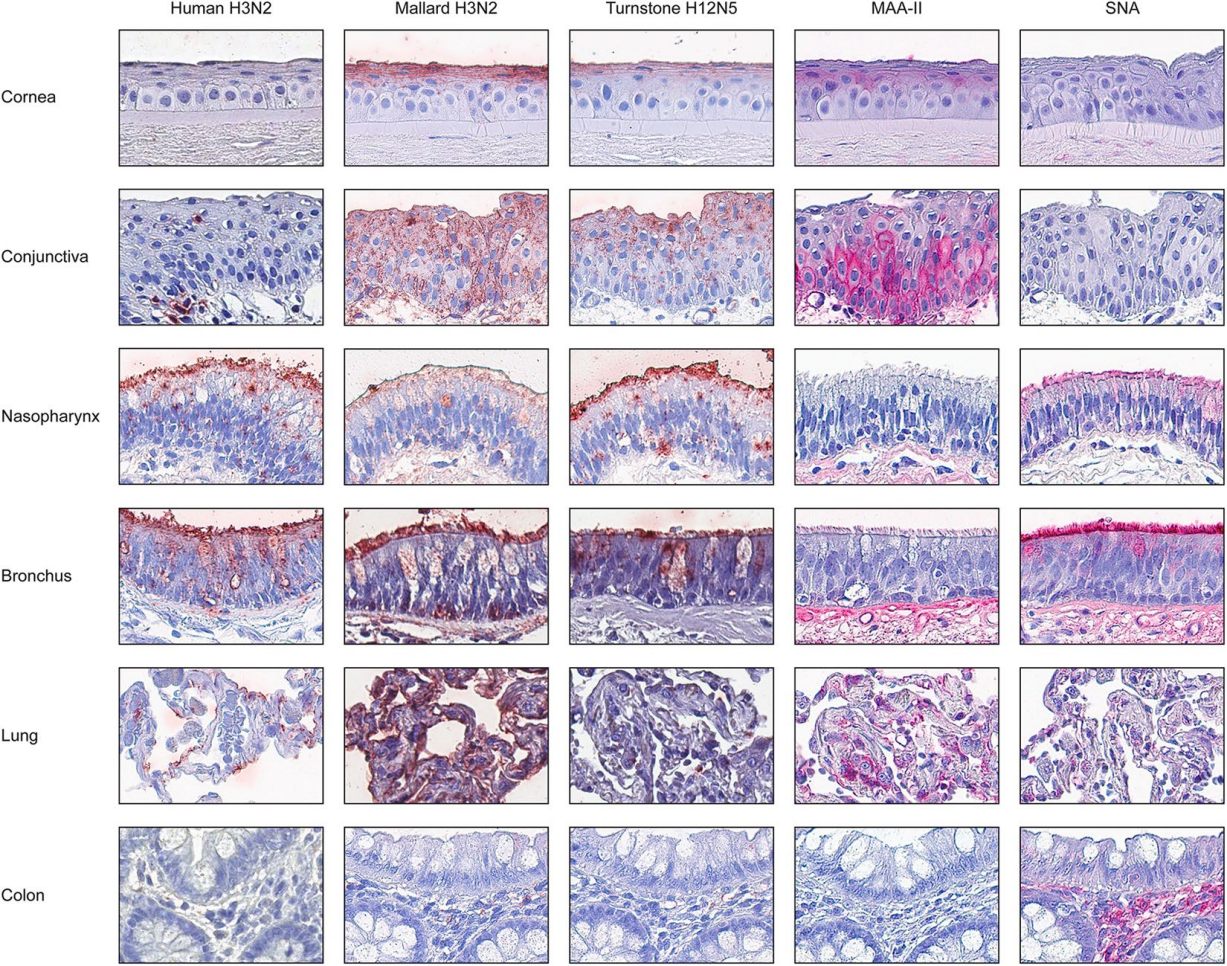
Attachment (in red) to human tissues of human H3N2, mallard H3N2, and ruddy turnstone H12N5 influenza viruses, as well as MAA-II and SNA lectins. The nuclei were counterstained with hematoxylin (blue). Panes showing human H3N2 virus staining of cornea, conjunctiva and colon were obtained from Lindskog *et al*.^23^ after agreement with the authors and included to enable direct comparison^23^.

All investigated IAVs attached to human tissues (Table 1). The control seasonal human H3N2 virus attached extensively to nasopharynx and bronchus, but weakly to lung alveoli. Extensive attachment was observed for the mallard H3N2 virus to most tested human tissues. The ruddy turnstone H12N5 virus showed widespread attachment to human conjunctiva and nasopharynx, but intermediate attachment to bronchus and very low attachment to lung alveoli. None of the investigated viruses attached to human colon. Intermediate MAA-II staining was observed in human eye and lung, but there was no staining of bronchus nor nasopharynx (Table 1). SNA staining was extensive in the human respiratory tract. No lectin staining was detected in human colon. Representative images of viral and lectin staining of human tissues are displayed in Fig. 2.

**Table 1:** Median scores of attachment of studied IAVs and lectins to human tissues.

Tissue	Cell type	Human H3N2		Mallard H3N2		Mallard H6N1		Turnstone H12N5		Gull H16N3		MAA-II		SNA	
		Score	n	Score	n	Score	n	Score	n	Score	n	Score	n	Score	n
Eye	Conjunctiva	−^a^	2^a^	+	3	+^a^	2^a^	+	3	+^a^	2^a^	+	1	−	1
	Cornea	−^a^	3^a^	+	4	+^a^	3^a^	+	3	+^a^	3^a^	±	1	−	1
Nasopharynx	Ciliated	+	4	+	9	+^a^	3^a^	+	9	+^a^	4^a^	−	8	+	8
	Goblet	+	4	+	7	+^a^	3^a^	+	7	+^a^	4^a^	−	8	+	7
Bronchus	Ciliated	+	4	+	5	+^a^	4^a^	±	5	+^a^	4^a^	−	2	+	2
	Goblet	+	4	+	4	+^a^	4^a^	±	4	+^a^	4^a^	−	2	+	1
Lung	Pneumocytes	+	4	+	10	±^a^	4^a^	±	10	+^a^	4^a^	±	7	+	7
	Macrophages	−	4	+	10	±^a^	4^a^	±	10	+^a^	4^a^	±	5	−	7
Colon	Epithelial	−^a^	4^a^	−	6	−^a^	4^a^	−	6	−^a^	4^a^	−	5	−	5
	Goblet	−^a^	4^a^	−	6	−^a^	4^a^	−	6	−^a^	4^a^	−	5	−	5
	Crypt	−^a^	4^a^	−	7	−^a^	4^a^	−	7	−^a^	4^a^	−	6	−	6

Scoring key: −: <1% stained cells, ±: 1–50% stained cells, and +: >50% stained cells. n - number of tested individuals.

^a^Values were obtained from Lindskog *et al*.^23^ after agreement with the authors and included to enable direct comparison^23^.

**Figure 3:**
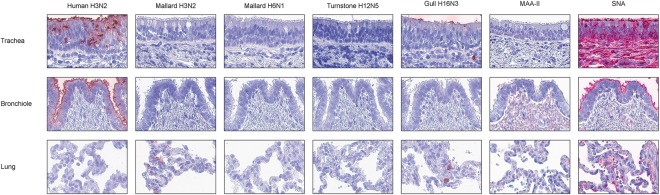
Attachment (in red) to pig tissues of human H3N2, mallard H3N2, mallard H6N1, ruddy turnstone H12N5, and black-headed gull H16N3 influenza viruses, as well as MAA-II and SNA lectins. The nuclei were counterstained with hematoxylin (blue).

Summaries of viral attachment to investigated pig tissues are shown in Table 2, with representative virus and lectin histochemistry images displayed in Fig. 3. The human H3N2 virus had intermediate attachment to pig trachea and strong attachment to pig bronchioles. Mallard H3N2 virus had intermediate attachment to pig alveoli. The mallard H6N1 and ruddy turnstone H12N5 viruses did not attach to any of the investigated pig tissues. Gull H16N3 virus attached only weakly to ciliated cells of pig trachea. No MAA-II signal was detected from pig respiratory tract (Table 2). Conversely, high levels of SNA staining were detected in pig upper and lower respiratory tracts.

**Table 2:** Median scores of attachment of studied IAVs and lectins to pig tissues.

Tissue	Cell type	Human H3N2		Mallard H3N2		Mallard H6N1		Turnstone H12N5		Gull H16N3		MAA-II		SNA	
		Score	n	Score	n	Score	n	Score	n	Score	n	Score	n	Score	n
Trachea	Ciliated	±	5	−	5	−	5	−	4	±	5	−	1	+	1
	Goblet	±	4	−	5	−	5	−	5	−	5	−	1	+	1
Bronchioles	Ciliated	+	2	−	4	−	4	−	2	−	4	−	2	+	2
	Goblet	+	2	−	4	−	4	−	2	−	4	−	2	+	1
Lung	Pneumocytes	−	2	±	4	−	4	−	2	−	4	−	2	±	2
	Macrophages	−	2	±	4	−	4	−	2	−	4	−	2	±	2

Scoring key: −: <1% staining, ±: 1–50% staining, +: >50% staining, and N/A: not applicable. n - number of tested individuals.

## Discussion

IAV transmission dynamics is characterized by a high amount of different possible subtypes circulating in wild birds^1,2^. The species barrier constituted by the difference in linkage conformation of the SA IAV receptor between humans and birds is well known^3,8,9^. Yet, reported human cases of LPAIV and HPAIV infections have repeatedly shown the possibility of direct IAV transmission between avian and human hosts^21,22^, and therefore, it is of importance to investigate the zoonotic potential of AIVs. The current study presents data comparing the attachment pattern to human and pig tissues, of a panel of avian viruses together with an evaluation of the distribution of different SA linkage conformations in the target tissues. In addition, the glycan receptor tropism of the analysed viruses was evaluated.

Charadriiformes has been described as an important source of AIVs complementary to Anseriformes, and ruddy turnstones (*Arenaria interpres*) are believed to play an important role in the dynamics of AIVs in North America^25,27^. Yet, there is very limited data available on receptor and tissue tropism of these viruses^14,15,23^. In this study, the ruddy turnstone H12N5 virus showed the highest attachment signal to cells of the human eye and nasopharynx, lower attachment intensity in the LRT tissues, and no observed attachment to human colon. The PVA of the H12N5 virus resembled that of gull H16N3 virus, suggesting the capacity of Charadriiformes viruses to attach to human eye and URT^23^. The PVA of the mallard H3N2 virus to cells of the human eye, nasopharynx, bronchus, and lung was extensive, and similar to earlier published observations of mallard H6N1 virus^10,23^. Glycan array analysis indicated broad, but selective, glycan tropism of both the mallard H3N2, H6N1 and ruddy turnstone H12N5 viruses. On the array, these three viruses attached both to avian and human receptor types, and to mixed structures, suggesting the propensity to attach to various human tissues. For the ruddy turnstone H12N5 virus, the attachment signal of Neu5Acα2,3Galβ1,4[6Su]GlcNAc was almost twice the attachment signal of Neu5Acα2,3[6Su]Galβ1,4GlcNAc, indicating the importance of the position of the sulfate group. There was no obvious multivalency effect observed for the oligoantennary 3′SLN N-glycans, since tetra-antennary 3′SLN N-glycan generally had lower attachment signal, probably due to steric hindrance. Similar to the mallard H6N1 and ruddy turnstone H12N5 viruses, the mallard H3N2 virus did not only show attachment to α2,3-linked SAs, but had extensive attachment to branched [6Su]Galβ1,3[Neu5Acα2,6]GalNAc, and weak attachment to 6′SdiLNs, which partly could explain the abundant attachment to human tissues. The control seasonal human H3N2 virus attached extensively to cells of the human upper airways in accordance with earlier studies^10,23^. Accordingly, lectin stainings showed very high levels of the α2,6-linked SA in the human upper airways. On the glycan array, the human H3N2 virus only attached to analogues of the 6′SLN structure with the highest attachment to 6′SdiLN, followed by 6Su-6′SLN, and 6′SLN. This is similar to observations by Kumari *et al*.^12^. The mallard H6N1 virus attached to a lesser number of different glycans compared to the mallard H3N2 virus, and attached mainly to α2,3-linked conformations of SA. The sulfated forms of the α2,3-linked SA structures had lower attachment, indicating that the sulfation was not beneficial for the H6N1 virus-glycan interaction. The mallard H6N1 virus had intense attachment signal to “chimeric” 3′S[Neu5Acα2,6]TF, and 3′STF. Approximately equal binding affinities for both 3′STF and 3′S[Neu5Acα2,6]TF have earlier been reported for a subset of duck IAVs^14^. These are mono- and disialylated forms of the O-glycan core 1 structure, indicating that these viruses may bind sialylated O-glycans as well. The two mallard viruses and the ruddy turnstone virus attached to [6Su]Galβ1-3[Neu5Acα2-6]GalNAc. The black-headed gull H16N3 virus had a very limited attachment to the array, only attaching to Neu5Acα2,3Galβ1,4[6Su]GlcNAc (i.e. a version of 6Su-3′SLN). Black-headed gull H16N3 virus has earlier been shown to attach overall strongly to several human tissues, including in the URT and LRT^23^. In this study, the gull H16N3 virus only attached weakly to pig trachea. Results from an earlier study by Gambaryan *et al*. suggest that SLe^x^ is the optimal receptor structure for gull viruses^14^. Unfortunately, there were no fucosylated structures incorporated in the glycan array used in the present study. However, the effect of the Fuc moiety in the study by Gambaryan *et al*. might not be pivotal for gull IAVs, since the reported binding affinities was similar for 3′SLN and SLe^x^ for the tested gull viruses^14^. As no H16 IAV was included in that study, no direct comparison can be made with the current virus panel used. In summary, the present study partly corroborates the findings from other studies of IAV receptor tropisms including main attachment to 3′SLN/3′STF, but also reveals new findings such as the unexpected attachment to several α2,6 sialylated structures. The results highlight the heterogeneity in glycan tropism between different IAV isolates. Still all viruses analysed only attached to glycans terminating in Neu5Ac and no attachment was observed to e.g. Neu5Gc or Kdn. Neu5Ac was thus a mutual receptor structure of the investigated IAVs, but the downstream carbohydrates and/or adjacent substituents had an important effect on the interaction, as demonstrated by the reported results in the present study^8^.

Seasonal influenza viruses usually infect the URT and give mild disease^10^. However, the deeper down in the respiratory tract a virus causes infection, the more severe the outcome might be^21,22^. Therefore, it may be worrisome from a pathogenicity perspective to find viruses that have an extensive attachment to cells of the LRT, as here reported for the mallard H3N2 virus and partly for the ruddy turnstone H12N5 virus. Together these observations provide further evidence of the ability of some AIVs circulating in wild birds to attach to human tissues, including the LRT.

The observed divergence in PVA between the human and mallard H3N2 viruses in pig bronchioles and lung are consistent with the reported preference for the human URT of human IAVs and the preference for the human LRT of mallard IAVs^10^. The mallard H6N1 virus used in this study has earlier been reported not to attach to pig trachea or bronchus, but displaying very weak attachment to pig bronchioles and attachment to a moderate number of type II pneumocytes^10^. In the current study, no attachment to any of the pig tissues, neither by the mallard H6N1 virus, nor the ruddy turnstone H12N5 virus was observed. Additionally, the black-headed gull H16N3 virus only attached weakly to ciliated cells of pig trachea. From the broad attachment patterns displayed on the glycan array, the mallard H6N1 and ruddy turnstone H12N5 viruses were both expected to attach to pig tissues. However, the lectin stainings did not detect any α2,3-linked SA in the investigated pig tissues, which may explain the low viral attachment observed to these tissues. The absence of detectable MAA-II staining in pig lung was unexpected, but sample duplicates of full sized lung sections were in accordance with the results from the tissue microarrays. More stainings of a larger set of pig tissues would be of interest to explore this further. Additionally, potential variance in glycan phenotype of epithelial cells of commercially available pigs from different breeds might add complexity to these types of studies.

As a general trend, the PVAs were weaker to pig tissues in contrast to the corresponding human tissues. The pig has been proposed as a “mixing vessel” for IAVs of various hosts^6–9^. The seminal paper by Scholtissek *et al*. first postulating the “mixing vessel” theory, is focusing on the role of the IAV nucleoprotein of H3N2 IAVs^6^. Ito *et al*. switched the focus towards the virus-cell attachment process and the host cell’s conformation of surface displayed SA by studying the linkage conformation of SA in duck colon and pig trachea^7^. However, recent studies based on both lectin staining and mass spectrometry analysis have reported high similarities between humans and pigs in regard to SA display in both respiratory and intestinal tissues^17,28,29^. In addition, both Byrd-Leotis *et al*. and Walther *et al*. report AIV attachment to both α2,3 and α2,6-linked SA, as well as to “chimeric” α2,3/α2,6-linked SA. Thus, observations in the current study of AIV attaching to both α2,3/α2,6-linked SA receptor types, as well as α2,3-linked SA structures being present in humans are supported by earlier studies.

In conclusion, the presented data corroborate the findings of other studies suggesting that AIV attachment is more extensive to human tissues in contrast to pig tissues, and that pigs display limited α2,3-linked SA in the respiratory tract^10,29^. Collectively, the results from these studies suggest that not all AIVs would require passage trough pigs to adapt to mammalian receptor structures. Yet, direct transmission of AIVs to humans is rare, while pigs have proven to be highly susceptible to AIV infection^3,5^. IAVs of subtypes H1N1 and H3N2 are widely circulating among pigs, where the former is classified as either “classical swine” or “avian-like” and the latter classified as either “avian-like” or “human-like”^5^. Yet, surveillance studies of wild birds have shown IAVs of hemagglutinin types H3, H4, and H6 to be the most frequently occurring hemagglutinin types in wild birds^1^. Hence, there remain several unanswered questions on what makes an AIV successfully cross the species barrier and jump from an avian to a mammalian host. Virus-host cell attachment (as determined by virus histochemistry) is a first important step to investigate the propensity of AIV infecting mammalian hosts. However, successful infection and replication is dependent on several additional cumulative factors apart from attachment alone. As exemplified by Kumari *et al*. the sole existence of α2,3 or α2,6-linked SA on the cell surface is not a warranty for a successful complete IAV replication cycle^12^. The existence of barriers preventing interspecies transmission of IAVs is well known^3,8,9^. However, in line with other studies, the reported results suggest that rather than the species barrier being determined by the receptor SA conformation alone, it comprises a combination of additional factors complementary to the receptor structure^2,6,17,28,29^. Such factors are e.g. the virus’ capacity of evading the host immune response and the ability to replicate efficiently in the infected host cell. Differences in e.g. host immune response and host cell biophysical properties might still require passage through pigs for an AIV to adapt to other mammalian hosts. Indeed, already Scholtissek *et al*. suggested that IAV host specificity is determined by several factors, and not a single factor^6^. Still, as mentioned earlier, there are many reports of infection and replication of AIV in human and pig cells, both *in vivo* and *in vitro*^21,30–32^. More extensive histochemistry studies of IAV on human vs. pig sections would be needed in order to fully validate the rationale of the pig as a “mixing vessel” for IAV of avian and mammalian (human) type. Such studies should ideally be complemented by infection studies in cell culture, using multiple cell lines and ideally primary cells representing the tissue of interest, to investigate the virus’ ability to replicate^30–33^. Finally, this study shows that AIV of various subtypes from diverse avian species have the capacity to both attach to α2,6-linked SA structures and have a broad capacity of attaching to human tissues. The zoonotic potential of AIVs can thus not be neglected.

## Material and Methods

### Ethics statement

Handling of human tissues was approved by the Uppsala Ethical Review Board (reference #2002-577). Human tissues were obtained from the Department of Pathology, Uppsala University Hospital, Uppsala, Sweden as part of the sample collection governed by the Uppsala Biobank (http://www.uppsalabiobank.uu.se/en). Human tissue donors had all given approved informed consent for non-commercial research use of tissue biopsies. All human tissue samples used in the present study were anonymised in accordance with approval and advisory report from the Uppsala Ethical Review Board. All experimental methods were performed in accordance with the relevant guidelines and regulations.

### Virus panel

Five different IAVs were FITC-labelled as previously described and investigated for their glycan and tissue tropisms using glycan array and virus histochemistry on human and pig tissue sections^10^. A human seasonal IAV, A/Netherlands/213/2003 (H3N2), was included as a reference^10,20,22,23^. Two AIVs isolated from mallards (Anseriformes; *Anas platyrhynchos*) were used; 1) A/Mallard/Sweden/68619/2007 (H3N2), of a subtype identical to the human isolate, and 2) A/Mallard/Sweden/81/2002 (H6N1), to investigate whether the mallard isolates would yield similar outcome, despite being of different subtypes^10,20,23^. Additionally, two different AIVs (A/Turnstone/Delaware/15/2007 (H12N5) and A/Black-headed gull/Sweden/2/1999 (H16N3)) isolated from Charadriiformes hosts were investigated^23^. Ruddy turnstones (*Arenaria interpres*) are thought to play an important part of the ecology of AIV in North America^27^. Additionally, H12 AIV has not been studied earlier using virus histochemistry and is interesting to compare to the PVA of gull H16N3 virus, since these viruses were isolated from two different host species, but belonging to the same order (Charadriiformes).

### Glycan array

A glycan array comprising 55 different glycans, (e.g. including different α2,3 and α2,6-linked SA structures) was used to investigate virus glycan attachment^34^. The structures of the glycans included in the array are displayed in the supplementary material Table S1. In brief, glass slides with immobilized glycans (each spotted as triplicates) were blocked with 50 mM ethanolamine, 50 mM sodiumtetraborate and 84 mM HCl (Sigma-Aldrich A/S, Brøndby, Denmark) followed by incubation on shake in a humidity chamber with 20 hemagglutinin units IAV together with 10 μM Oseltamivir (Sigma-Aldrich) per well at room temperature for one hour, reagents were diluted in 1 × PBS-T (Sigma-Aldrich). After washing with 1 × PBS-T (Sigma-Aldrich) for three times, viruses were detected by α-FITC rabbit polyclonal antibody (#ab19492, Abcam, Cambridge, U.K.) in combination with Alexa555-labeled α-rabbit IgG polyclonal antibody (#ab150078, Abcam). Negative controls without virus were included for determination of any unspecific signal. The fluorescence signal was measured by a Scan Array G_x_ Microarray Scanner (PerkinElmer A/S, Skovlunde, Denmark) and analysed by ProScanArray Express Version 4.0 (PerkinElmer), Microsoft Excel 14.6.9 (Microsoft AB, Kista, Sweden) and RStudio 1.0.136 (RStudio Core Team (2016), Vienna, Austria).

### Tissue preparation

Human tissue specimens were obtained from the Department of Pathology at the Uppsala University Hospital, Sweden, directed by the Uppsala Biobank (www.uppsalabiobank.uu.se/en/). Hematoxylin and eosin stained slides of each paraffin-embedded tissue were reviewed by a pathologist in order to obtain histologically normal, non-malignant tissue material without lesions or signs of infections, although each tissue was originally sampled due to other reasons not related to the present investigation. All tissue samples were anonymous following the Uppsala Ethical Review Board (Reference #2002-577), and available tissue information is therefore limited to age, gender and original diagnosis based on histology. All nasopharynx tissue samples were obtained by nasal polyp removal, while the bronchus and lung samples belonged to individuals with lung cancer, except for one lung sample obtained from an individual with metastatic melanoma. The colon tissue samples were derived from two individuals with colorectal cancer, one individual with metastatic endometroid cancer, two individuals with adenoma, one individual with diverticulosis, and one individual were only normal histology was noted in the sample. Eye tissues belonged to two individuals with melanoma and one individual with oligodendroglioma. Only tissue specimens determined as histologically normal, non-malignant by the pathologist were used in the present study.

Pig respiratory tissues were obtained from a small abattoir in central Sweden after passing slaughterhouse routine veterinary examination. The pigs were of Pigham crossbreed (sow: native breed x Yorkshire, boar: Hampshire x Duroc) and eight months of age at slaughter. The tissues were dissected from each individual immediately after slaughter and put into 4% formaldehyde solution. The tissues were paraffin embedded at the Department of Pathology, Uppsala University Hospital, similar to the treatment of the human tissues.

In brief, formalin-fixed and paraffin-embedded human and pig tissue specimens were used for generation of tissue microarrays (TMAs), as well as sections in full size format. In the supplementary material Tables S2–S5, a complete list of the number of individuals stained in the study is provided, as well as the distribution of full sections vs. TMA format. TMAs were generated as described previously^35^, using 1 mm tissue cores. Four μm thick sections for TMAs and full size slides were prepared from tissue blocks using a microtome (HM 355 S, Microm, Thermo Scientific, Waltham, MA, U.S.A.). Sections were mounted on adhesive slides (SuperFrost Plus, Thermo Scientific) and baked for 45 minutes at 60 °C prior to virus histochemistry.

### Virus histochemistry

Virus attachment was studied by virus histochemistry^10^. In brief, tissue slides were deparaffinised with xylene, hydrated in graded alcohols to distilled water, and blocked for endogenous peroxidase in 0.3% hydrogen peroxide. Each tissue section was incubated overnight at 4 °C with 50 hemagglutinin units of purified formalin fixed FITC-labelled IAV or 1 × PBS (Medicago AB, Uppsala, Sweden) as negative control. FITC-labelled viruses were detected by a peroxidase labelled α-FITC rabbit polyclonal antibody (#ab19492, Abcam). The signal was amplified by a tyramide signal amplification kit (PerkinElmer AB, Upplands Väsby, Sweden). Peroxidase signal was revealed with 3-amino-9-ethyl-carbazole (Sigma-Aldrich AB, Stockholm, Sweden). Tissues were counterstained with hematoxylin (Sigma-Aldrich), mounted with Vision Mount (Thermo Fisher Scientific) and scanned using Aperio Image Scope (Aperio Technologies, CA. U.S.A.) using a 20x objective. Two independent observers scored all digital virus histochemistry images. The percentage of stained cells of a given cell type in each tissue was scored according to a 3-tiered scale: “−” (<1% stained cells), “±” (1–50% stained cells), or “+” (>50% stained cells).

### Lectin stainings

To visualize the linkage conformation of SA in the investigated tissues, each tissue type was stained with MAA-II binding to α2,3-linked SA and SNA binding to α2,6-linked SA. Tissue sections were stained with either 4 μg/mL MAA-II (BioNordika AB, Stockholm, Sweden) or 2 μg/mL SNA (BioNordika) from Vector Laboratories. Bound lectins were detected by the Vectastain ABC-AP kit (BioNordika) together with the ImmPACT Vector Red Alkaline Phosphatase Substrate Kit (BioNordika). Lectin stained tissue specimens were counterstained, mounted, scanned, and scored following the protocol as described in the virus histochemistry paragraph.

### Data availability statement

The authors confirm that the summarized data supporting the findings of this study are available within the article and its supplementary materials. The raw experimental data set is available from the corresponding author, PEl, upon request.

## Electronic supplementary material

Supplementary tables
