# Supplementary material for: Characterization of avian influenza virus attachment patterns to human and pig tissues
Source: Sci Rep. 2018 Aug 15;8:12215. doi: 10.1038/s41598-018-29578-1 (PMC6093914; doi:10.1038/s41598-018-29578-1)
Supplement: Supplementary file 1 — Supplementary tables [file 41598_2018_29578_MOESM1_ESM.pdf]

Supplementary material

Characterization of avian influenza virus attachment patterns to human and pig tissues

Per Eriksson[1†], Cecilia Lindskog[2†], Ebbe Engholm[3], Ola Blixt[3], Jonas Waldenström[4], Vincent Munster[5], Åke Lundkvist[1], Björn Olsen[6], Elsa Jourdain[7] & Patrik Ellström[6*]

Affiliations

1. Zoonosis Science Centre, Department of Medical Biochemistry and Microbiology (IMBIM), Uppsala University, Uppsala SE-751 23, Sweden

2. Department of Immunology, Genetics and Pathology, Science for Life Laboratory, Uppsala University, Uppsala SE-751 85, Sweden

3. Department of Chemistry, Chemical Biology, University of Copenhagen, Frederiksberg C DK-1871, Denmark

4. Centre for Ecology and Evolution in Microbial Model Systems, Linnaeus University, Kalmar SE-392 31, Sweden

5. Laboratory of Virology, Rocky Mountain Laboratories, National Institute of Allergy and Infectious Diseases, National Institutes of Health, Hamilton, Montana 59840, U.S.A.

6. Zoonosis Science Centre, Department of Medical Sciences, Uppsala University, Uppsala SE-751 23, Sweden

7. UMR0346 - EPIA, INRA, VetAgro Sup, Saint Genès Champanelle FR-63122, France.

†These authors contributed equally to the study.

Table S1. Glycan structures in the glycan array with normalized attachment (%) of each virus. The attachment signals were normalized towards the glycan with the highest virus attachment for each tested virus. Only normalized values with a value >3 times larger than baseline are shown. HH3N2 – human H3N2, MH3N2 – mallard H3N2, MH6N1 – mallard H6N1, TH12N5 – ruddy turnstone H12N5, and GH16N3 – black-headed gull H16N3 influenza viruses.

	Glycan number	Glycan structure	Abbreviation[1]	HH3N2	MH3N2	MH6N1	TH12N5	GH16N3
No SA	1	GalNAcαsp	Tn					
	2	Galβ1-3GalNAcαsp	TF					
	3	Galβ1-4Glcβsp	Lac					
	4	Galβ1-4GlcNAcβsp	LN					
	5	Galβ1-4GlcNAc-Bi-antennary N-glycan	LN bi-antennary N-glycan					
3 Su	6	[3Su]Galβ1-4GlcNAcβsp	3Su-LN					
	7	[3Su]Galβ1-3GalNAcβsp	3Su-TF					
	8	[3Su]Galβ1-3GlcNAcβsp	3Su-Le^c					
6 Su	9	[6Su]Galβ1-4GlcNAcβsp	6Su-LN					
	10	[6Su]Galβ1-3GlcNAcβsp	6Su-Le^c					
	11	[6Su]Galβ1-4[6Su]GlcNAcβsp	di-6Su-LN					
	12	[6Su]Galβ1-3[6Su]GlcNAcβsp	di-6Su-Le^c					
	13	[6Su]Galβ1-3GalNAcαsp	6Su-TF					
Di/tri Su	14	[3Su]Galβ1-4[6Su]GlcNAcβsp	3,6Su-LN					
	15	[3,6diSu]Galβ1-4GlcNAcβsp	3,6diSu-LN					
	16	[4,6diSu]Galβ1-4GlcNAcβsp	4,6diSu-LN					
	17	[4,6diSu]Galβ1-4GlcNAcβsp3	4,6diSu-LN					
	18	[3,6diSu]Galβ1-4[6Su]GlcNAcβsp	3,6triSu-LN					
SA	19	Neu5Acαsp	Neu5Ac					
	20	Neu5Gcαsp	Neu5Gc					
	21	[9OAc]-Neu5Acsp	9OAc-Neu5Ac					
α2,3 SA	22	Neu5Acα2-3Galβsp	"avian receptor"		90	31	76	
	23	Neu5Acα2-3Galβ1,4Glcβsp	3'SL		99	77	75	
	24	Neu5Acα2-3Galβ1,4GlcNAcβsp	3'SLN		61	86	73	
	25	Neu5Acα2-3Galβ1,3GalNAcαsp	3'STF		77	65	83	
	26	Neu5Acα2-3[6Su]Galβ1,4GlcNAcβsp	6Su-3'SLN		75	6	48	

	27	KDNα2-3Galβ1-4GlcNAcβsp	Kdn-LN					
	28	Neu5Acα2-3Galβ1-4[6Su]GlcNAcβsp	6Su-3'SLN		97	28	90	100
	29	GalNAcβ1-4[Neu5Acα2-3]Galβ1-4Glcβsp						
	30	Neu5Acα2-8Neu5Acα2-3Galβ1-4Glcβsp	3'diSL					
	31	GalNAcβ1-4[Neu5Acα2-8Neu5Acα2-3]Galβ1-4Glcβsp						
	32	Neu5Acα2-8Neu5Acα2-8Neu5Acα2-3Galβ1-4Glcβsp	3'triSL					
	33	GalNAcβ1-4[Neu5Acα2-8Neu5Acα2-8Neu5Acα2-3]Galβ1-4Glcβsp						
	34	Neu5Acα2-3Galβ1-4GlcNAc-Bi-antennary N-glycan	3'SLN bi-antennary N-glycan		100	80	82	
	35	Neu5Acα2-3Galβ1-4GlcNAc-Tri(I)-antennary N-glycan	3'SLN tri(I)-antenanry N-glycan		100	100	98	
	36	Neu5Acα2-3Galβ1-4GlcNAc-Tri(II)-antennary N-glycan	3'SLN tri(II)-antennary N-glycan		100	89	97	
	37	Neu5Acα2-3Galβ1-4GlcNAc-Tetra-antennary N-glycan	3'SLN tetra-antennary N-glycan		92	15	75	
α2,6 SA	38	Neu5Acα2-6GalNAcαsp	"human receptor"					
	39	Neu5Acα2-6Galβsp	"human receptor"					
	40	Neu5Acα2-6Galβ1-4Glcβsp	6'SL					
	41	Neu5Acα2-6Galβ1-4GlcNAcβsp	6'SLN	31				
	42	Neu5Acα2-6Galβ1-4GlcNAcβ1-3Galβ1-4GlcNAcβsp	6'SdiLN	100	5			
	43	Neu5Acα2-6Galβ1-3GalNAcαsp	6'STF					
	44	Neu5Gcα2-6Galβ1-4GlcNAcβsp	6'Neu5Gc-LN					
	45	[9OAc]Neu5Acα2-6Galβ1-4GlcNAcβsp	9OAc-6'SLN					
	46	[6Su]Galβ1-3[Neu5Acα2-6]GalNAcαsp			86	47	85	
	47	Neu5Acα2-6Galβ1-4[6Su]GlcNAcβsp	6Su-6'SLN	38				
	48	Galβ1-3[NeuAcα2-6]GlcNAcβ1-3Galβ1-4Glcβsp						
	49	Neu5Acα2-6Galβ1-4GlcNAc-Bi-antennary N-glycan	6'SLN bi-antennary N-glycan					
	50	Neu5Acα2-6Galβ1-4GlcNAc-Tri(I)-antennary N-glycan	6'SLN tri(I)-antennary N-glycan		5			

	51	Neu5Acα2-6Galβ1-4GlcNAc-Tri(II)-antennary N-glycan	6'SLN tri(II)-antennary N-glycan		3			
	52	Neu5Acα2-6Galβ1-4GlcNAc-Tetra-antennary N-glycan	6'SLN tetra-antennary N-glycan					
oligo SA	53	Neu5Acα2-3Galβ1-3[Neu5Acα2-6]GalNAcαsp	3'S[Neu5Acα 2-6]TF			84	100	
	54	Neu5Acα2-8Neu5Acαsp	diNeu5Ac					
	55	Neu5Acα2-8Neu5Acα2-8Neu5Acαsp	triNeu5Ac					

1. Abbreviations where applicable.

Table S2. Number of virus stained human individuals in full section vs. TMA format. N/A – not applicable.

	Human H3N2		Mallard H3N2		Turnstone H12N5	
**Tissue**	**Full section**	**TMA**	**Full section**	**TMA**	**Full section**	**TMA**
**Eye**	N/A	N/A	3	1	3	N/A
**Nasopharynx**	4	N/A	4	5	4	5
**Bronchus**	4	N/A	4	1	4	1
**Alveoli**	4	N/A	4	6	4	6
**Colon**	N/A	N/A	N/A	7	N/A	7

Table S3. Number of lectin stained human individuals in full section vs. TMA format. N/A – not applicable.

	MAA-II		SNA	
**Tissue**	**Full section**	**TMA**	**Full section**	**TMA**
**Eye**	1[1]	N/A	1[1]	N/A
**Nasopharynx**	2	7	2	6
**Bronchus**	1	1	1	1
**Alveoli**	1	6	1	6
**Colon**	N/A	6	N/A	6

1. Repeated staining with the same individual performed.

Table S4. Number of viral stained pig individuals in full section vs. TMA format. N/A – not applicable.

	Human H3N2		Mallard H3N2		Mallard H6N1		Turnstone H12N5		Gull H16N3	
**Tissue**	**Full section**	**TMA**	**Full section**	**TMA**	**Full section**	**TMA**	**Full section**	**TMA**	**Full section**	**TMA**
**Trachea**	N/A	5	N/A	5	N/A	5	N/A	5	N/A	5
**Bronchiole**	2	N/A	4	N/A	4	N/A	2	N/A	4	N/A
**Alveoli**	2	N/A	4	N/A	4	N/A	2	N/A	4	N/A

Table S5. Number of lectin stained pig individuals in full section vs. TMA format. N/A – not applicable.

	MAA-II		SNA	
**Tissue**	**Full section**	**TMA**	**Full section**	**TMA**
**Trachea**	1	N/A	1	N/A
**Bronchiole**	2[1]	N/A	2	N/A
**Alveoli**	2[1]	N/A	2	N/A

1. Repeated staining with the same individuals performed.
